# Effects of hydrolyzed yeast supplementation on growth performance, immunity, antioxidant capacity, and microbial shedding in weaning pigs

**DOI:** 10.14202/vetworld.2020.1902-1909

**Published:** 2020-09-18

**Authors:** Waewaree Boontiam, Chalong Wachirapakorn, Phreerapong Phaengphairee

**Affiliations:** Department of Animal Science, Faculty of Agriculture, Khon Kaen University, Khon Kaen 40002, Thailand

**Keywords:** antioxidant capacity, growth performance, hydrolyzed yeast, immunity, weaning pigs

## Abstract

**Background and Aim:**

Weaning pigs normally suffer from many stressors which have impaired growth performance and immunity. Hydrolyzed yeast has been proposed as an alternative feed additive. The aim of this study was to investigate the effects of various levels of hydrolyzed yeast (HY) supplementation in the feed of weaning pigs on growth performance, diarrhea incidence, immunity, antioxidant capacity, and microbial populations.

**Materials and Methods:**

A total of 144 crossbred weaning pigs (Duroc × Landrace × Large White) with a mean body weight (BW) of 7.46 kg were randomly assigned to one of four treatments during a 5-week feeding trial. Treatments consisted of a basal diet without HY inclusion (control), or the basal diet supplemented with HY at 0.5, 1.0, and 1.5 g/kg of diet, respectively.

**Results:**

Piglets fed with 1.0 or 1.5 g/kg HY presented significantly increased BW (p=0.009) and decreased incidence of diarrhea (p=0.001). The final BW (p=0.012), average daily gain (p=0.094), and average daily feed intake (p=0.091) showed a linear improvement with the level of HY inclusion. However, the gain-to-feed ratio was unaffected by dietary treatments. Linear responses to the HY supplementation levels were also observed for blood urea nitrogen (p=0.030), total protein (p=0.017), lymphocyte percentage (p=0.064), catalase activity (p=0.089), malondialdehyde (MDA) level (p=0.001), *Salmonella* spp. (p=0.024), *Escherichia coli* (p=0.021), and *Lactobacillus* spp. (p=0.048). Dietary inclusion of HY at 1.0 and 1.5 g/kg resulted in increased immunoglobulin A and G secretions (p=0.042 and p=0.022, respectively) and decreased MDA concentration (p<0.01) and *Salmonella* spp. (p=0.026) and *E. coli* (p=0.050).

**Conclusion:**

It was concluded that HY inclusion at 1.0 and 1.5 g/kg in the diet of weaning pigs improve BW, immunoglobulin secretion, and antioxidant enzyme activity, whereas it lowers diarrhea occurrence, lipid peroxidation, and pathogenic bacteria in weaning pigs.

## Introduction

Post-weaning diarrhea (PWD) is of critical concern in swine production worldwide and can typically result in lower feed intake, growth retardation, and immune suppression [1]. A previous report has established that *Escherichia coli* is the predominant pathogenic strain causing PWD in piglets [2]. During colonization of the epithelial cells of the small intestine by the *E. coli* cells, it produces one or more enterotoxins that induce increased gut permeability, histological damage, and cytokine production [3], leading to increased morbidity and mortality in piglets. Numerous feed-based approaches have been used to investigate the modulatory effects of certain feed supplements on gut health and/or immunity in weaning pigs to improve disease resistance and growth performance [4,5].

Hydrolyzed yeast (HY) is an end product of yeast extraction through hydrolysis, and it typically contains both yeast extract and yeast cell walls. Yeast cells are composed of mannan-oligosaccharides (MOS), and β-glucans, and their byproducts are known for their highly digestible protein, nucleotide, and amino acid (glutamic acid) contents [6]. The previous report showed that pigs fed with yeast-derived products exhibited recovery of intestinal integrity and immune responses after incidences of diarrhea [6]. The positive functions of yeast extract or yeast cells in modulating growth performance, immunity, balanced intestinal microbiota, and antioxidant capability of weaning pigs have also been documented [6-8]. Furthermore, improvements in intestinal mucosal structure and nutrient digestibility were reported for weaning pigs fed 1.2 g/kg of a mixture of yeast cells and cell walls [6]. Similarly, growth performance and populations of beneficial bacteria improved with diets supplemented with 2 g/kg of HY, in lactating sows [9]. Moreover, Tuoi *et al*. [10] reported that the percentage incidence of diarrhea and *E. coli* shedding decreased in piglets that were fed 1 g/kg of yeast-derivative product.

The aim of this study was to investigate the effects of various levels of HY supplementation in the feed of weaning pigs on growth performance, incidence of diarrhea, immunity, antioxidant capacity, and microbial populations.

## Materials and Methods

### Ethical approval

All animal procedures used in this study were reviewed and approved by the Animal Care and Use Committee of Khon Kaen University (IACUC-KKU77/61).

### Study period and location

This study was conducted from March to April 2019 at the University Farm of Khon Kaen University, Thailand.

### Animals, diet, and management

A total of 144 piglets [Duroc × Landrace × Large White] weaned at 28 days of age with mean body weight (BW) of 7.46 kg were grouped in blocks by BW and gender and were randomly allotted one of four dietary treatments in a randomized complete block design. Each treatment consisted of six replicates, with six piglets (three gilts and three barrows) per pen. The weaning pigs were fed diets supplemented with various levels of HY for 5 weeks. The control basal diet did not include HY (Group 1), while the remaining groups were fed a basal diet with HY inclusion at 0.5 (Group 2, HY5), 1.0 (Group 3, HY10), and 1.5 (Group 4, HY15) g/kg of diet. The main active ingredients in HY were crude protein (40%), free nucleotides (3.5%), β-glucans (23%), MOS (15%), and glutamic acid (4.9%). The HY supplements were delivered as a top-dressing on the basal diet and prepared weekly to ensure feed freshness. The nutrient composition was calculated to meet or exceed National Research Council recommendation [11], as shown in Table-1. A mash diet was randomly chosen and kept in a sealed sample box for proximate analyses of crude protein, ether extract, and ash contents [12].

**Table-1:** Feed ingredients and nutrient composition of the basal diet (% as fed basis).

Ingredient	% as fed basis
Broken rice	52.16
Full-fat soybean	20.00
Soybean meal (44% CP)	14.36
Fish meal (58% CP)	5.00
Skim milk	5.00
Dicalcium phosphate	1.30
Monocalcium phosphate	1.15
Sodium chloride	0.33
L-lysine monochloride (98%)	0.09
DL-methionine	0.31
L-threonine	0.05
Vitamin-mineral premix^1^	0.25
Total	100.00
Calculated composition	
Crude protein (%)	22.00
Metabolizable energy, kcal/kg	3,450
Lysine (%)	1.41
Methionine (%)	0.68
Threonine (%)	0.92
Tryptophan (%)	0.27
Calcium (%)	1.01
Total phosphorus (%)	1.04
Analyzed composition	
Crude protein (%)	19.78
Ether extract (%)	4.97
Ash (%)	5.03

1 Supplied (per kilogram diet): Vitamin A as retinol, 8,400 IU; Vitamin D_3_, 945 IU; Vitamin E, 0.0126 g; Vitamin K, 0.0021 g; Vitamin B_1_ (thiamine), 0.0011 g; Vitamin B_2_ (riboflavin), 0.0022 g; Vitamin B_6_ (pyridoxine), 0.0016 g; Vitamin B_12_ (cyanocobalamin), 0.02 mg; nicotinic acid, 0.0126 g; pantothenic acid, 0.063 g; folic acid, 0.0053 g; biotin, 0.0315 mg; choline, 0.175g; copper as CuSO_4_, 0.126 g; iron as FeSO_4_, 0.105 g; manganese, 0.021 g; cobalt, 0.0007 g; iodine, 0.0007 g; selenium as Na_2_SeO_3_, 0.00007 g

During the experiment, the piglets were maintained in pens (0.96 m×2.16 m, with a stocking density of 0.35 m^2^ per pig) with half-slatted concrete floors and in an open-housed environment. The temperature in the experimental house ranged from 20 to 23°C between 21:00 and 06:00 and from 28 to 32°C between 09:00 and 17:00, during the experimental period. Each pen contained a hemp sack, and a heating lamp at night during the 2-week post-weaning period to ensure the required temperature for the piglets. The diets were administered to the pigs 3 times daily at 06:00, 12:00, and 17:00. All piglets had *ad libitum* access to the feed and drinking water through a self-feeder and nipple drinker, respectively, throughout the experimental period.

### Growth performance

BW and feed intake were monitored on weeks 0, 2, and 5 post-weaning, and also used for evaluating average daily gain (ADG) and average daily feed intake (ADFI) evaluations. Per pen, the ADG and ADFI were calculated by dividing the total weight gain and total feed intake, respectively, by the total number of experimental days. The gain-to-feed ratio (G:F) was calculated for each pig by dividing the ADG by the ADFI.

### Diarrheal score

During post-weaning days 1-28, the number of diarrheic piglets per pen was monitored every morning at 06:00. Diarrheal occurrence was defined by the feces being soft and its moisture content over 75%. Diarrheal rate (%) was calculated by [total number of diarrheic piglets/(total number of piglets × days of experiment)] × 100 [13].

### Blood collection and analysis

Blood was collected on day 35 after overnight fasting using six healthy piglets (three barrows and three gilts, total n = 24) per treatment. Blood (5 mL) was collected through an anterior vena cava puncture, using disposable syringes with needles. Blood samples (3 mL) were immediately transferred to a BD Vacutainer (Becton Dickinson Vacutainer System, Franklin Lakes, NJ, USA) with non-anticoagulant. All samples were kept at room temperature for 2 h and subsequently centrifuged for 10 min at 3000*g* to separate the serum. The representative serum was used for further analyses of metabolic profiles, immunoglobulin concentrations, and antioxidant capacity.

Concentrations of aspartate aminotransferase (AST), blood urea nitrogen (BUN), and total protein were measured using commercial kits (Boehringer Mannheim, Germany), and their values were detected using an automatic blood analyzer. Antioxidant enzyme activity for catalase (CAT), glutathione peroxidase (GSH-Px), and malondialdehyde (MDA) was quantified using a spectrophotometer, following the manufacturer’s instructions (Nanjing Jiancheng Bioengineering Institute, Nanjing, China). The immunoglobulin concentration was assayed with porcine immunoglobulin A (IgA) and immunoglobulin G (IgG) using Enzyme-linked immune-sorbent assay quantitation kits (Bethyl Laboratories, Inc., Montgomery, TX, USA). Each sample was analyzed in triplicate with 10 and 100 thousand-fold dilutions for the IgA and IgG assays, respectively. The absorbance of each sampling plate was detected within 30 min at 450 nm using an automatic microplate reader (Thermo Lab Systems, Finland). Values were presented as mg/mL. Aliquots (2 mL) of blood samples were transferred into heparinized tubes for assessing leukocyte percentages [14]. All blood-related criteria were analyzed in duplicate.

### Microbial shedding

On day 35, approximately 2 g of fresh feces were directly sampled from the rectum of the piglets using manual stimulation of the internal and external sphincters. Fecal samples were diluted ten-fold in a sterile saline solution (0.9%, Becton, Dickinson and Co., Franklin Lakes, NJ, USA). Plates for *Salmonella* spp. (using *Salmonella-Shigella* agar) were incubated under anaerobic conditions at 37°C for 48 h. The count of *E. coli* was enumerated in MacConkey agar for 24 h at 37°C. *Lactobacillus* spp. was isolated on Rogosa and Sharpe agar with an overnight incubation (20-24 h) [15]. Typical colonies of each bacterium on each agar were immediately counted after removing from the incubator. The counts of microbial populations were log-transformed before statistical analysis was carried out. The results were presented as log_10_ colony-forming units/g.

### Statistical analysis

All data were analyzed in a randomized complete block design using PROC GLM model in the SAS statistical software package (SAS University Edition). Each pen was an experimental unit for growth performance and diarrheal rate, whereas individually selected piglet was an experimental unit for blood metabolites and microbial counts. The recorded value of the log_10_ microbial count was transformed before analysis, and normality was verified with the Kolmogorov–Smirnov test. Significant differences among dietary treatments were evaluated by Duncan’s new multiple range test at a probability value of p≤0.05, whereas a tendency was assumed at a probability value between p>0.05 and p<0.10.

## Results

### Growth performance and diarrheal occurrence

The ADG, ADFI, and G:F ratio of the weaning pigs that were fed HY was unaffected by dietary treatment (Table-2). However, compared to the control, BW significantly improved in the HY-supplemented groups at 5 weeks (p=0.009). Linear improvements in BW (p=0.012), ADG (p=0.094), and ADFI (p=0.091) were observed with increasing levels of HY inclusion. In addition, the piglets fed 1.0 and 1.5 g/kg of HY had a significantly lower percentage of diarrheal incidence from 14 to 28 days than that in the control (p=0.002). Furthermore, the lower diarrheal rate was linearly influenced by increasing HY supplementation levels across all periods (p=0.009, p<0.001, and p=0.014, respectively).

**Table-2:** Effect of dietary hydrolyzed yeast supplementation on growth performance and diarrhea occurrence in weaning pigs^1^.

Criteria	Hydrolyzed yeast supplementation				SEM	Probability		
	
	Control	HY5	HY10	HY15		Treatment	Linear	Quadratic
BW (kg)								
Initial	7.33	7.57	7.53	7.42	0.440	0.979	0.880	0.966
2^nd^ week	10.26	11.34	11.64	11.80	0.582	0.273	0.116	0.134
5^th^ week	17.77^B^	19.84^A^	20.06^A^	19.77^A^	0.454	0.009	0.012	0.028
ADG (g)								
0-2 week	209.29	269.29	293.57	312.86	30.365	0.127	0.050	0.051
3-5 week	357.62	404.76	400.95	379.52	33.068	0.733	0.647	0.794
Overall	298.29	350.57	358.00	352.86	19.621	0.152	0.094	0.134
ADFI (g)								
0-2 week	356	377	386	396	22.551	0.645	0.286	0.301
3-5 week	690	789	778	768	30.436	0.130	0.091	0.287
Overall	549	574	574	543	21.496	0.636	0.855	0.888
G/F ratio								
0-2 week	0.59	0.71	0.76	0.79	0.102	0.641	0.347	0.272
3-5 week	0.52	0.51	0.52	0.50	0.047	0.970	0.728	0.933
Overall	0.55	0.61	0.62	0.65	0.045	0.411	0.174	0.171
Diarrhea rate (%)							
0-2 week	5.04^a^	4.26^a,b^	3.36^b^	3.10^b^	0.500	0.057	0.009	0.318
3-4 week	4.48^A^	3.34^A,B^	2.94^B^	2.66^B^	0.282	0.002	<0.001	0.024
Overall	6.61^a^	5.19^a,b^	4.83^b^	4.43^b^	0.553	0.066	0.014	0.120

HY5, HY10 and HY15 = Basal diet with hydrolyzed yeast supplementation at 0.5 1.0 and 1.5 g/kg diet, respectively.

1 The pigs were fed the experimental diet from an average initial BW of 7.46 kg to a final BW of 19.36 kg.

a,b Means within the same row with different letters show a significant difference of p<0.05.

A,B Means within the same row with different letters show a significant difference of p<0.01. BW=Body weight, ADG=Average daily gain, ADFI=Average daily feed intake, SEM=Standard error of the mean

### Blood profiles

Dietary HY supplementation did not seem to affect the AST, BUN, and total protein concentrations (Table-3). However, a linear relationship was noted between increasing levels of HY and decreasing BUN concentrations (p=0.030) as well as increasing total protein concentration (p=0.017).

**Table-3:** Effect of dietary hydrolyzed yeast supplementation on blood profiles in weaning pigs^1^.

Criteria	Hydrolyzed yeast supplementation				SEM	Probability		
	
	Control	HY5	HY10	HY15		Treatment	Linear	Quadratic
AST (U/L)	50.56	47.02	49.14	57.73	8.374	0.981	0882	0.955
BUN (mmol/L)	6.56	4.02	3.53	3.89	0.795	0.061	0.030	0.190
Total protein (g/L)	43.83	59.28	71.07	68.49	7.174	0.067	0.017	0.384

HY5, HY10 and HY15=Basal diet with hydrolyzed yeast supplementation at 0.5 1.0 and 1.5 g/kg diet, respectively.

1 Least squares means of six piglets per treatment. AST=Aspartate aminotransferase, BUN=Blood urea nitrogen, SEM=Standard error of the mean

### Immunoglobulins and leukocytes

The IgA and IgG concentrations increased significantly with HY supplementation at 1.5 g/kg compared to that of the control diet (p=0.042, p=0.022, respectively) (Table-4). Increasing HY inclusion also enhanced IgA and IgG secretions, both of which showed linear responses (p=0.009, p=0.003, respectively). Furthermore, HY inclusion elicited a significant linear improvement in lymphocyte percentage (p=0.064). However, the leukocyte percentages of neutrophils, monocytes, and eosinophils were unaffected across dietary treatments.

**Table-4:** Effect of dietary hydrolyzed yeast supplementation on secretory immunoglobulins and leukocyte counts in weaning pigs^1^.

Criteria	Hydrolyzedyeast supplementation				SEM	Probability		
	
	Control	HY5	HY10	HY15		Treatment	Linear	Quadratic
Immunoglobulin								
IgA (mg/mL)	0.56^b^	0.88^a,b^	0.95^a^	1.03^a^	0.111	0.042	0.009	0.102
IgG (mg/mL)	0.39^B^	0.54^A,B^	0.66^A,B^	0.73^A^	0.070	0.022	0.003	0.142
Leukocytes count (%)								
Lymphocytes	49.17	61.83	67.50	71.67	8.166	0.272	0.064	0.319
Neutrophils	39.33	33.02	29.57	30.66	4.009	0.348	0.121	0.525
Monocytes	1.29	1.37	1.26	1.29	0.272	0.993	0.926	0.854
Eosinophils	0.66	0.57	0.67	0.64	0.199	0.984	0.983	0.758

HY5, HY10 and HY15=Basal diet with hydrolyzed yeast supplementation at 0.5 1.0 and 1.5 g/kg diet, respectively.

1 Least squares means of six piglets per treatment.

a,b Means within the same row with different letters show a significant difference of p<0.05.

A,B Means within the same row with different letters show a significant difference of p<0.01. IgA=Immunoglobulin A, IgG=Immunoglobulin G, SEM=Standard error of the mean

### Antioxidant enzyme and lipid peroxidation

Lower MDA concentration was found in the HY-supplemented groups than that the control (p<0.001). In addition, increasing levels of HY linearly and quadratically affected the concentrations of CAT (p=0.089, p=0.074, respectively) and MDA (p*<*0.001, p=0.022, respectively) (Table-5). No significant difference was detected in GSH-Px concentration.

**Table-5:** Effect of dietary hydrolyzed yeast supplementation on serum antioxidant status in weaning pigs^1^.

Criteria	Hydrolyzed yeast supplementation				SEM	Probability		
	
	Control	HY5	HY10	HY15		Treatment	Linear	Quadratic
CAT (U/mL)	2.54	4.42	3.94	4.23	0.565	0.121	0.089	0.074
GSH-Px (U/mL)	303.57	370.69	394.33	384.32	51.483	0.606	0.266	0.587
MDA (nm/mL)	7.29^A^	4.76^B^	3.41^B^	2.97^B^	0.578	<0.001	<0.001	0.022

HY5, HY10 and HY15=Basal diet with hydrolyzed yeast supplementation at 0.5 1.0 and 1.5 g/kg diet, respectively.

A,B Means within the same row with different letters show a significant difference of p<0.01. CAT=Catalase, GSH-Px=Glutathione peroxidase, MDA=Malondialdehyde.

1 Least squares means of six piglets per treatment, SEM=Standard error of the mean

### Fecal microbial shedding

Dietary HY supplementation reduced the population of *Salmonella* spp. (p=0.024, p=0.016) and *E. coli* (p=0.021, p=0.019) for the linear and quadratic responses, respectively, whereas the population of *Lactobacillus* spp. increased linearly with HY supplementation level (p=0.048; Table-6). The population of *Salmonella* spp. was lower with HY treatment than that with the control (p=0.026). Furthermore, the 1.0 and 1.5 g/kg HY inclusion levels significantly decreased *E. coli* shedding in the piglet feces compared to that of the control (p=0.050).

**Table-6:** Effect of dietary hydrolyzed yeast supplementation on fecal microbial shedding in weaning pigs^1^.

Criteria	Hydrolyzed yeast supplementation				SEM	Probability		
	
	Control	HY5	HY10	HY15		Treatment	Linear	Quadratic
Microbial shedding, log_10_ cfu/g								
*Salmonella* spp.	6.67^a^	4.23^b^	4.94^b^	4.36^b^	0.553	0.026	0.024	0.016
*Escherichia coli*	5.94^a^	4.18^b^	4.75^a,b^	3.65^b^	0.546	0.050	0.021	0.019
*Lactobacillus* spp.	6.22	8.28	9.09	9.32	1.049	0.189	0.048	0.293

HY5, HY10 and HY15=Basal diet with hydrolyzed yeast supplementation at 0.5, 1.0 and 1.5 g/kg diet, respectively.

1 Least squares means of six piglets per treatment.

a,b Means within the same row with different letters show a significant difference of p<0.05. SEM=Standard error of the mean

## Discussion

Piglets fed HY grew faster than those that were fed the control diet. These findings are consistent with the results of Hu *et al*. [16], who observed significant increase in BW at 28 days in weaning pigs that were fed yeast-derived protein. Our study also found that increased HY levels induced ADFI in the piglets. The stimulation of feed intake found in this study was possibly influenced by the presence of glutamic acid and nucleotides in the yeast extract. The previous studies have used glutamic acid and nucleotides as flavor enhancers [17,18], since they play an important role in stimulating the exocrine secretion of saliva and gastric and pancreatic juices, as well as binding to taste cell receptors in the oral cavity. This mechanism allows taste nerves in the umami taste region to be activated [17]. However, no improvement was observed in the G:F ratio with HY supplementation, as was also observed by Santos *et al*. [19]. Our data indicated that HY inclusion at up to 1.5 g/kg of diet increased the BW of the piglets without affecting growth performance.

PWD results from an infection in the small intestine that inhibits the digestion and absorption capacity, with a consequent increase in the number of pathogenic bacteria in the large intestine [20]. In our study, the incidence of PWD decreased throughout the experimental period with the HY treatment, which corroborates the finding of Tuoi *et al*. [10], who observed lower incidence of diarrhea in piglets receiving a diet supplemented with a 1 g/kg mixture of β-glucan and MOS. The lower occurrence of diarrhea in HY-fed animals may have been due to the reduced proliferation of enterotoxigenic bacteria in the gastrointestinal tract, thus resulting in greater suppression of pathogenic bacteria by the modulation of beneficial microbiota [21]. Although, HY can be used as a partial protein source for animals, high crude protein inclusion in diet has been reported to increase the rates of diarrhea in piglets due to high amounts of undigested proteins passing through the large intestine [22]. Pathogenic bacteria can use these substances for cell proliferation that subsequently leads to PWD in the piglets [22]. This is consistent with the quadratic response to the HY supplementation level from 3 to 4 weeks, although there was no effect observed for the overall period. One explanation for this result may be that the lower BUN concentration could perhaps increase protein utilization in piglets that were fed HY.

In our study, the positive effects of HY included a reduction in the abundance of pathogenic bacteria and lower incidence of diarrhea. Together, these results imply that HY supplementation restores healthy functioning of the gastrointestinal tract of piglets, during the post-weaning period.

### Metabolic profiles

Blood metabolites can indicate the metabolic status of animals. Our results indicated an improvement in the total protein concentration in weaned pigs that were fed diets supplemented with HY, which is consistent with a previous study that used a yeast product as a partial alternative protein source for high protein availability in circulating blood [22]. Furthermore, dietary HY supplementation led to a significant decrease in the BUN concentration, indicating a high utilization of the yeast protein source by the weaned piglets, with less excretion of nitrogenous compound [23]. In this study, the increased rate of protein utilization led to a reduction in the level of undigested nitrogen, resulting in reduced proliferation of pathogenic bacteria in the feces of the weaned pigs. However, the AST concentration was unchanged by the HY-supplemented diets, indicating that the increased HY level did not result in damaged hepatocytes.

### Immunoglobulins and leukocytes

Serum immunoglobulin levels can be used as an indicator of the humoral immune response in animals. Changes in the levels of these proteins can affect animal productivity and immunity. Both IgA and IgG are important for defense against pathogenic invasion [24,25]; IgA is abundant in breast milk and modulates piglet immunity through the gastric and intestinal mucosa [24], while IgG is abundant in the bloodstream [25]. This study found that piglets receiving HY-supplemented diets exhibited a greater production of IgA and IgG than those that fed on the control diet. Our results agree with those of the previous study [26], where it was shown that the contents of β-glucans, MOS, nucleotides, or small peptides in a HY-supplemented group contributed to activating immunoglobulin secretion. Wang *et al*. [26] showed that the inclusion of small peptides at 3 g/kg increased increased immunoglobulin secretions in piglets. Furthermore, the presence of β-glucan in yeast cell walls has been shown to stimulate cytokine production and intestinal lymphocytes through modulation of Peyer’s patches as a result of the activation of mucosal immunity [27]. Xiong *et al*. [6] have also reported a reduction in the number of neutrophil granulocytes with β-glucan–enriched diets. Although this positive effect has been confirmed in a previous study [28], it is in contrast with our finding. The different results obtained among the studies might have been influenced by the dosage of β-glucan, diet composition, or the status of individual piglets. In this study, stimulation of the immune system was detected as an increase in IgA and IgG concentrations, which would be important in defending against pathogenic bacteria through secondary immune response.

### Antioxidant enzyme and lipid peroxidation

CAT and GSH-Px are important antioxidant enzymes in preventing cellular damage and maintaining cellular structure. Reduced concentrations of these enzymes lead to increased MDA secretion that can result in DNA damage [29,30]. Our study demonstrated that weaning pigs that were fed with HY could defend against free radicals through increased CAT activity. In agreement with this, Salobir *et al*. [31] showed that yeast-derived nucleotides can alleviate oxidative stress in piglets. In addition, the HY used in this study had a relatively greater quantity of glutamine that was included as an active precursor for glutathione biosynthesis. Published research has established the important function of glutathione in alleviating the effects of reactive oxygen species [30]. However, we did not observe an increase in GSH-Px activity with HY supplementation, which may have been due to a higher CAT level than that of GSH-Px, normally found in hepatocytes. In addition, CAT may have initially converted hydrogen peroxide to water and oxygen before being eliminated by the activity of GSH-Px, resulting in reduced MDA production, as has been previously observed [32].

### Fecal microbial shedding

The gut microbiota are important for the maintenance of gut homeostasis in animals [33]. We observed that potential components of the yeast cell wall or yeast extract present in HY, stimulate beneficial bacteria, immune responses, and protect against pathogens in the digesta. In our study, the populations of *Salmonella* spp. and *E. coli* decreased, while that of *Lactobacillus* spp. increased with HY supplementation. This finding is in agreement with that of Tuoi *et al*. [10], who observed that inclusion of combinations of 1.0 g/kg β-glucan and MOS in the piglet diet markedly increased the abundance of *Lactobacillus* spp. However, this contrasted with a previous report in which an increase in intestinal microbiota was not observed [34]. These inconsistent results may be due to the different inclusion levels, dietary compositions, or HY purity utilized. β-Glucan and MOS are reported to be active non-starch polysaccharides that likely promote the expansion of *Lactobacillus* and *Bifidobacteria* populations [10,16] and reduce those of *E. coli* and *Salmonella* spp. in weaning pigs [35]. The regulation of gut microbiota found in our study was supported by the increased lymphocyte percentages and concentrations of secreted IgA and IgG. This approach could be applied in feed formulations for piglets to balance their gut microbiota during the weaning period.

## Conclusion

Piglets fed with HY at 1.0 and 1.5 g/kg of diet showed significantly improved BW and immunoglobulin secretion, as well as decreased diarrheal occurrence, MDA concentration, and populations of *E. coli* and *Salmonella* spp.

## Authors’ Contributions

WB designed and performed the experiment, collected the data, interpreted data, wrote and revised the manuscript. CW supervised the study. PP performed the statistical analysis. All authors read and approved the final manuscript.
